# Complexity of *Brassica oleracea*–*Alternaria brassicicola* Susceptible Interaction Reveals Downregulation of Photosynthesis at Ultrastructural, Transcriptional, and Physiological Levels

**DOI:** 10.3390/cells9102329

**Published:** 2020-10-20

**Authors:** Violetta Katarzyna Macioszek, Magdalena Gapińska, Agnieszka Zmienko, Mirosław Sobczak, Andrzej Skoczowski, Jakub Oliwa, Andrzej Kiejstut Kononowicz

**Affiliations:** 1 Laboratory of Plant Physiology, Department of Biology and Plant Ecology, Faculty of Biology, University of Bialystok, 15-245 Bialystok, Poland; 2 Laboratory of Microscopy Imaging and Specialized Biological Techniques, Faculty of Biology and Environmental Protection, University of Lodz, 90-237 Lodz, Poland; magdalena.gapinska@biol.uni.lodz.pl; 3 Department of Molecular and Systems Biology, Institute of Bioorganic Chemistry, Polish Academy of Sciences, 61-704 Poznan, Poland; akisiel@ibch.poznan.pl; 4 Department of Botany, Institute of Biology, Warsaw University of Life Sciences (SGGW), 02-787 Warsaw, Poland; miroslaw_sobczak@sggw.edu.pl; 5 Institute of Biology, Pedagogical University in Krakow, 30-084 Krakow, Poland; andrzej.skoczowski@up.krakow.pl; 6 Department of Chemistry and Biochemistry, Institute of Basic Sciences, University of Physical Education in Krakow, 31-571 Krakow, Poland; jakub.oliwa@gmail.com; 7 Department of Plant Ecophysiology, Faculty of Biology and Environmental Protection, University of Lodz, 90-237 Lodz, Poland; andrzej.kononowicz@biol.uni.lodz.pl

**Keywords:** *Alternaria brassicicola*, chlorophyll a fluorescence, chloroplast ultrastructure, defense response, microarray, photosynthesis, susceptibility

## Abstract

Black spot disease, caused by *Alternaria brassicicola* in *Brassica* species, is one of the most devastating diseases all over the world, especially since there is no known fully resistant *Brassica* cultivar. In this study, the visualization of black spot disease development on *Brassica oleracea* var. *capitata* f. *alba* (white cabbage) leaves and subsequent ultrastructural, molecular and physiological investigations were conducted. Inter- and intracellular hyphae growth within leaf tissues led to the loss of host cell integrity and various levels of organelle disintegration. Severe symptoms of chloroplast damage included the degeneration of chloroplast envelope and grana, and the loss of electron denseness by stroma at the advanced stage of infection. Transcriptional profiling of infected leaves revealed that photosynthesis was the most negatively regulated biological process. However, in infected leaves, chlorophyll and carotenoid content did not decrease until 48 hpi, and several chlorophyll *a* fluorescence parameters, such as photosystem II quantum yield (F_v_/F_m_), non-photochemical quenching (NPQ), or plant vitality parameter (Rdf) decreased significantly at 24 and 48 hpi compared to control leaves. Our results indicate that the initial stages of interaction between *B. oleracea* and *A. brassicicola* are not uniform within an inoculation site and show a complexity of host responses and fungal attempts to overcome host cell defense mechanisms. The downregulation of photosynthesis at the early stage of this susceptible interaction suggests that it may be a part of a host defense strategy, or, alternatively, that chloroplasts are targets for the unknown virulence factor(s) of *A. brassicicola*. However, the observed decrease of photosynthetic efficiency at the later stages of infection is a result of the fungus-induced necrotic lesion expansion.

## 1. Introduction

*Brassica oleracea* var. *capitata* (head cabbage) from the *Brassicaceae* family is one of eight *Brassica* subspecies and an important vegetable in the human diet due to its nutritional values [1]. Many white, red, and Savoy cabbage cultivars are cultivated widely in Europe, Asia, and North America, and used as a staple diet item, an ingredient of many national cuisine vegetable dishes or an addition to salads. In Northern Europe and New Zealand, cabbage is often used as feed for sheep and cattle [1,2]. As a widespread crop, cabbage is exposed to many bacterial and fungal diseases, which lower its yield all over the world. One of the most devastating fungus-induced diseases to all Brassicas is black spot disease, caused by *Alternaria brassicicola*. Cabbage cultivars show various levels of susceptibility to *A. brassicicola*, ranging from moderately to highly susceptible, but there is still not any known fully resistant cabbage cultivar [3]. 

*Alternaria brassicicola* belongs to the large fungal division ascomycota (*Ascomycetes*), order *Pleosporales*. According to the novel taxonomic classification based on the molecular phylogenetic analysis, genus *Alternaria* consists of 27 sections with a distinct Brassicicola one [4]. As a necrotrophic fungus, *A. brassicicola* is seed-transmitted, and thus infects young seedlings causing their damping off [5,6]. In the case of mature plants, the fungus is wind- and insect-spread, infecting preferentially older leaves. This results in black/brownish spreading lesions, which, at advanced stages of infection, can be responsible for the decay of the whole plant [7,8,9]. As a post-harvest pathogen, *A. brassicicola* has been found on cabbage debris and cabbage heads during storage [10]. The infection cycle of *A. brassicicola* is simple, from a single conidium to a mycelial network, often with overlapping stages typical for necrotrophic fungi [11]. After the initial attachment of the conidia to the leaf surface and germination, the fungus penetrates the host tissues through the appressoria and stomata, or invades them directly, using a preferential mode of penetration (depending on *Brassica* cultivar), or all of them simultaneously [3,11,12]. The fungus forms dome-shaped and usually aseptate appressoria at germ tube tips, similar to appressoria formed by *Botrytis cinerea*. Humidity, temperature, and conidial concentration are factors that influence *A. brassicicola* germination and infection of a host under field and laboratory conditions [13,14]. Prior to and during colonization, the fungus actively kills host cells primarily through the secretion of lipases, cell wall degrading enzymes, small secondary metabolites and was recently identified as the most abundant phytotoxin in *A. brassicicola* cultures—brassicicolin A [15,16,17]. Many *A. brassicicola* mutants with a depleted production of these secreted compounds, which also exhibit different levels of germination inhibition, were tested *in planta*, showing various degrees of virulence inhibition [16]. However, a primary virulence factor of *A. brassicicola* has not been discovered to date, thus it remains unknown how this fungus kills host plant cells. Moreover, it has been postulated that the possible resistance against *A. brassicicola* in Brassicas might be based on the cumulative effect of several genes rather than monogenicity [18,19].

The knowledge concerning plant defense mechanisms against *A. brassicicola* infection is mostly based on the extensively investigated model pathosystem for *Brassicaceae*—non-host *Arabidopsis thaliana* and *A. brassicicola*. Arabidopsis resistance against the fungus is compromised in *pad3* and *coi1* mutants, indicating that it requires camalexin and jasmonic acid (JA)-dependent signaling, respectively [20]. The hypersensitive host cell death during this interaction is restricted to the inoculation site, and accompanied by generation of reactive oxygen species (ROS) and callose deposition in wild-type Arabidopsis Col-0 [21,22]. On the other hand, camalexin is not metabolized by the fungus and inhibits its germination and development, but Brassicas phytoalexins induced during interaction with *A. brassicicola*, in most cases, are detoxified [23,24,25]. Although the transcriptome profiling of Arabidopsis Col-0 during *A. brassicicola* infection indicates that the photosynthesis-related genes remain unaffected in the resistant interaction [26]; Zmienko and Macioszek, unpublished data], the microarray analysis of a susceptible *pad3* mutant infected with *A. brassicicola* has demonstrated downregulation of photosynthesis-related genes [27]. Moreover, recent research on susceptible *Brassica juncea* infected with *A. brassicicola* revealed severe changes in chloroplast ultrastructure, and a post-inoculation time- and leaf position-dependent decrease in chlorophyll *a*:*b* ratio and photosynthesis efficiency [9].

Here, we report on the susceptible interaction between *B. oleracea* var. *capitata* f. *alba* (cultivar ‘Glory of Enkhuizen’) and *A. brassicicola*, both from the fungus and host plant perspective. In our work, we focused on the details of the fungal development and colony formation and the plant cell reactions during infection, at both light and transmission microscopy levels. Moreover, analyses of changes in the host transcriptional profiles with a special attention paid to photosynthesis-related genes and related physiological host responses to the fungus were conducted.

## 2. Materials and Methods

### 2.1. Plant Growth, Fungal Strain, and Inoculation

The seeds of *Brassica oleracea* var. *capitat*a f. *alba* (white cabbage) early cultivar ‘Glory of Enkhuizen’ were obtained from a Polish seed company and grown in soil:perlite mixture (15:1) in a plant growth room under fluorescent light (Super TLD Philips 865) at 100 µmol m^−2^ s^−1^, 16 h day/8 h night photoperiod, at temperature 22 ± 1 °C and relative humidity of approximately 65%. The wild type strain of *A. brassicicola* (ATTC 96836) was grown on potato dextrose agar plates (PDA; Difco, the Netherlands) for 7–10 days under the same conditions as plants, but in the dark. The second leaves of four-leaf mature *B. oleracea* plants were inoculated with the *A. brassicicola* conidial suspension at a concentration of 5 × 10^5^ conidia per ml of distilled water, or just distilled water in case of control plants, by putting one or two 10 µL drops per leaf or spraying the leaves using a Nalgene aerosol spray bottle (Sigma-Aldrich, St. Louis, MO, USA) [9,11]. In all experiments, plants were inoculated 4-6 h after switching on the light (about 10.00 a.m.–12.00 a.m. local time). The inoculated plants were incubated in transparent plastic boxes to maintain high humidity under the same light and temperature conditions as for growing. 

### 2.2. Disease Progression Analysis

*Brassica oleracea* plants were drop-inoculated, and necrotic spot parameters such as area, perimeter and average radius were measured using the WinDIAS Leaf Image Analysis System (Delta-T Devices, Cambridge, UK). For each time point, the second leaf of at least 6 plants was detached and images were taken immediately at 24, 48 and 72 h post-inoculation (hpi) in 3 independent experiments (*n* = 3). 

### 2.3. Light Microscopy

To investigate the stages of *A. brassicicola* development during leaf infection, aniline blue-lactophenol (Sigma-Aldrich, St. Louis, MO, USA) stain was applied on drop-inoculated leaves at 4, 8, 12, 14, 16, 20, and 24 hpi [11]. The numbers of germinated conidia, germ tubes, and appressoria were counted per 100 conidia at random sites of the inoculation area on the second leaf of 3 plants per each time point. 

### 2.4. Scanning and Transmission Electron Microscopy 

To investigate the changes of leaf surface and fungal growth during infection, 1.5 cm diameter leaf discs were cut out from the *B. oleracea* second leaves sprayed or drop-inoculated with the conidial suspension. The samples were examined under a tabletop scanning electron microscope TM-1000 (SEM; Hitachi, Tokyo, Japan) operating at 15 kV, without any pre-processing [11]. Samples from six plants per experiment were harvested at 12, 24, 48, and 72 hpi. The experiment was repeated independently twice. 

For the ultrastructural investigations, samples from the second leaf of the control and drop-inoculated plants were collected at 48 hpi and embedded in Epon–Spurr resin mixture [9]. The semi-thin (1 µm thick) and ultra-thin sections (80 nm thick) were obtained using a Reichert Jung ultramicrotome (Leica, Germany). The ultra-thin sections, after staining with uranyl acetate and lead citrate [28], were examined under a transmission electron microscope JEOL 1010 (TEM; JEOL, Japan) operating at 80 kV. The ultrastructure of the plant cells was observed on at least 50 micrographs per each treatment. 

### 2.5. Microarray Experiment

The time-course pattern of gene expression was examined in the second leaves of *B. oleracea* sprayed with *A. brassicicola* conidial suspension by cDNA hybridization to 29 k Arabidopsis Oligonucleotide Microarrays (University of Arizona) (GEO platform GPL7725). Each sample treated as a single biological replicate was collected from 3 control or 3 infected plants. For each time point (0, 12, 24, and 48 hpi), 2 biological replicates were analyzed and a common reference design was applied, where each sample was labeled with Cy5 and hybridized against the Cy3-labeled pool of the control plants. 

Each microarray probe sequence (~70-mer) was used as a query in a BLASTN search against *Brassica* nucleotide sequences available in the GenBank database. The best hit was recorded for each probe. Probes with high homology to *Brassica* targets were selected by applying a Bit score cutoff value of 40. For 66% of them, probe:target alignments were >60 nt in length and displayed >75% sequence identity.

The total RNA was extracted with RNeasy Plant Mini Kit (Qiagen, Hilden, Germany) and samples were DNase-digested with a TURBO DNA-free kit (Ambion, Austin, TX, USA). The sample quality was analyzed with a Nanodrop 1000 spectrophotometer (Thermo Scientific, Miami, OK, USA) and 2100 Bioanalyzer (Agilent Technologies, Wood Dale, IL, USA). For each labeling reaction, 15 µg of high-quality RNA (A_260_/A*280* and A_260_/A_230_ ≥ 2, RIN ≥ 9) was reverse-transcribed using the SuperScript Indirect cDNA Labeling System (Invitrogen, Waltham, MA, USA) according to the manufacturer’s protocol. Aminoallyl-modified cDNA was coupled with 5 μL of Cy3 or Cy5 ester dye (Amersham Pharmacia, Buckinghamshire, UK) and unbound dye was removed with a MinElute Reaction Cleanup Kit (Qiagen, Hilden, Germany). The efficiency of the labeling was monitored with NanoDrop measurements. Corresponding Cy3 and Cy5-cDNA were mixed, the volume was adjusted to 10 µL and the samples were heat-denatured at 95 °C for 10 min and added to 115 µL pre-warmed (68 °C) hybridization buffer SlideHyb #3 (Ambion, Austin, TX, USA). Hybridization was carried out in a HybArray 12 workstation (PerkinElmer, Hebron, KY, USA) at 42 °C for 18 h. The microarrays were washed with 2× SSC (Ambion, Austin, TX, USA) supplemented with 0.1% SDS (Sigma-Aldrich, St. Louis, MO, USA) at 42 °C, 0.5× SSC at 30 °C and 0.005× SSC at 25 °C each for 5 cycles (20 s flow, 40 s hold) and dried by centrifuging. Images were collected with a ScanArray Express scanner (PerkinElmer, Hebron, KY, USA) at 100% laser intensity and variable PMT settings.

The images were analyzed with GenePix 6.1 software (Molecular Devices, San Jose, CA, USA) using the morphological opening background subtraction method with default parameters. The data were further analyzed in an R/Bioconductor environment using the limma package [29,30,31]. Foreground and background intensities were read with “genepix.custom” function. After background subtraction and printtip-loss normalization within arrays, followed by scaling, the log-ratios to have the same median-absolute-deviation (MAD) across arrays, a linear model was constructed for the contrasts of interest. Differential expression of genes for which the probes passed homology-based filtering criteria (see above) was assessed by moderated t-statistics and correcting for multiple testing using the false discovery rate (FDR) [32]. The adjusted *p*-value 0.1 was chosen as a significance threshold. 

For functional analysis and gene annotation, *A. thaliana* gene IDs originally assigned to the microarray probes were used. Only 8014 genes with high homology to *Brassica* were taken into consideration and used as a background in the Gene Ontology (GO) and MapMan analyses.

A GO enrichment analysis of differentially regulated genes was performed separately for each time point against the list of all genes represented on the microarray using the ThaleMine v.4.1.1 online analysis tool [33]. The *p*-value was calculated using a hypergeometric distribution test and adjusted with the Holm–Bonferroni multiple test correction, and the significance threshold was set at a *p*-value < 0.05. The visualization of significantly regulated GO terms was performed using REVIGO [34], which reduces long lists of Gene Ontology terms by summarizing and removing redundant GO terms. The input data were prepared as follows. From the list of all microarray results (Table S1), genes differentially expressed at 48 hpi (FDR < 0.1) were selected. The GO_cellular_process terms and the log fold change expression values of these genes were used as an input for REVIGO. Genes without assigned GO_cellular_process terms as well as genes for which GO: 0008150 (biological_process_unknown) was the only assigned GO term, were excluded from the analysis, resulting in an input list of 275 genes. The output list of REVIGO-summarized GO terms is presented in Table S2 and visualized in Figure 7. The MapMan (v.3.5.0) analysis was performed based on the probe mapping files for *A. thaliana* and pathways downloaded from the MapMan repository [35]. 

### 2.6. Photosynthetic Pigment Content

Chlorophyll was extracted in 100% methanol and measured using a spectrophotometer PowerWave XP (BioTek, Crawfordsville, IN, USA). The content of chlorophyll *a*, chlorophyll *b*, and carotenoids was calculated according to Wellburn [36]. The samples were harvested from 3 control and 3 infected plants per time point, and each experiment was repeated independently 3 times (*n* = 3). 

### 2.7. Chlorophyll a Fluorescence

The control and drop-inoculated infected plants were dark-adapted for 30 min, then each second leaf was detached and immediately subjected to analysis of the kinetics of chlorophyll a fluorescence quenching using a Handy FluorCam 1000-H System (Photon Systems Instruments, Czech Republic) according to the manufacturer’s built-in protocol. The measurements were performed on the selected area (diameter about 1.5 cm) of control untreated leaves, as well as infected ones containing necrosis and adjacent tissue. The duration of the reading procedure per one leaf was 4 min, and parameters such as F_0_ (minimum fluorescence), F_m_ (maximum fluorescence), F_v_ (variable fluorescence) as well as F_p_ (peak fluorescence during the initial phase of Kautsky effect) were measured accordingly during 4 light adaptation (L1–L4), steady-state in light (Lss) and/or 3 dark relaxation periods (D1–D3). Based on the measured chlorophyll *a* fluorescence parameters, automatic calculation of over 45 parameters such as QYmax (maximum photosystem II quantum yield, F_V_/F_M_), F_v_/F_m_ (photosystem II quantum yield), F_q_/F_v_ (photosystem II efficiency factor), Rfd (fluorescence decline ratio) and qL (fraction of photosystem II open centers) in light adaptation periods and steady-state in light was performed and NPQ (non-photochemical quenching) and qP (photochemical quenching of variable fluorescence) were also calculated in the dark relaxation periods. All the measured and calculated chlorophyll a fluorescence parameters are shown in Table S6. In each experiment, 3–4 control and 3–4 infected plants were used per time point. The experiment was repeated independently 3 times (*n* = 3).

### 2.8. Statistical Analysis

The statistical analysis of all the data obtained in this work, except the microarray data, was performed using analysis of variance (ANOVA) and post-hoc Duncan’s test (*p* < 0.05) using STATISTICA v.13.3.

All the figures were composed using Adobe Photoshop or Corel Software.

## 3. Results

### 3.1. Black Spot Disease Development

**Figure 1:**
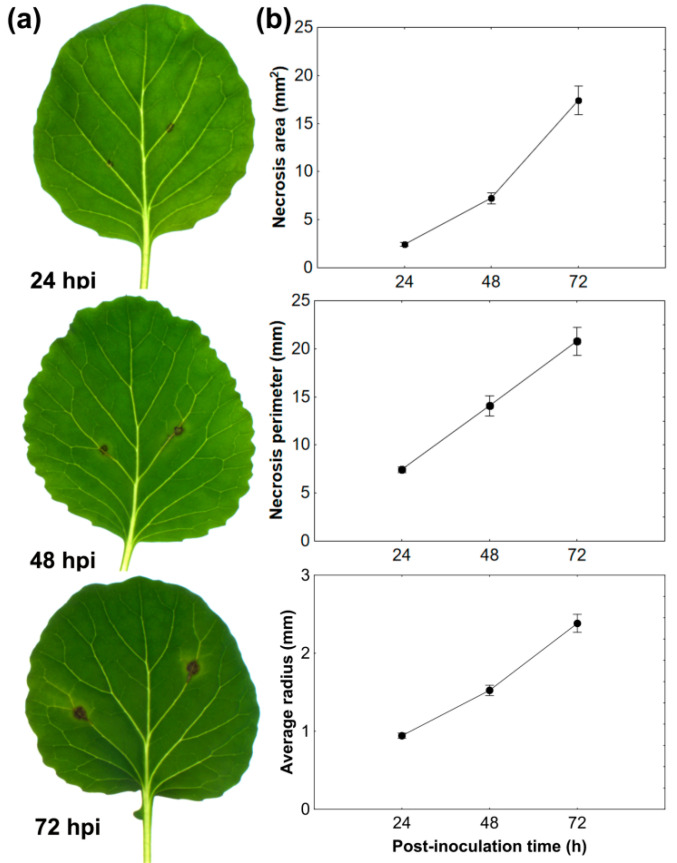
Analyses of necrotic lesion development on the second fully developed leaf of *B. oleracea* infected with *A. brassicicola*: (**a**) time-course images of necrosis development on the drop-inoculated leaves; (**b**) quantitative analysis of necrotic spot size changes. Data were gained using the WinDIAS system. The means ± SE were calculated from at least 18 measurements per time point obtained in 3 independent experiments (*n* = 3).

The areas infected with A. brassicicola became macroscopically visible in the form of small brown necrotic spots on the second leaf of *B. oleracea* plants at 20–24 hpi. The round-shaped necroses expanded beyond the inoculation sites at 48 and 72 hpi, and they were surrounded by a discrete chlorotic ring (Figure 1a). Necrosis parameters such as area, perimeter, and average radius increased in a time-dependent manner, indicating fast and massive fungal development on the susceptible host (Figure 1b). Inoculation of all four fully developed leaves of *B. oleracea* plants revealed that the size of the necrotic spots was leaf position-dependent, the older the leaf, the larger the necrosis, both on *in planta* and detached leaves assays (Figure S1).

### 3.2. A. brassicicola Development on Leaf Surface and Host Cell Responses

The appearance of macroscopically visible necrotic spots at the inoculation sites on *B. oleracea* leaves at 24 hpi was strictly correlated with the overlapping stages of the *A. brassicicola* infection cycle, such as pre-penetration, penetration, colonization, and conidiation, which started before this time point.

**Figure 2:**
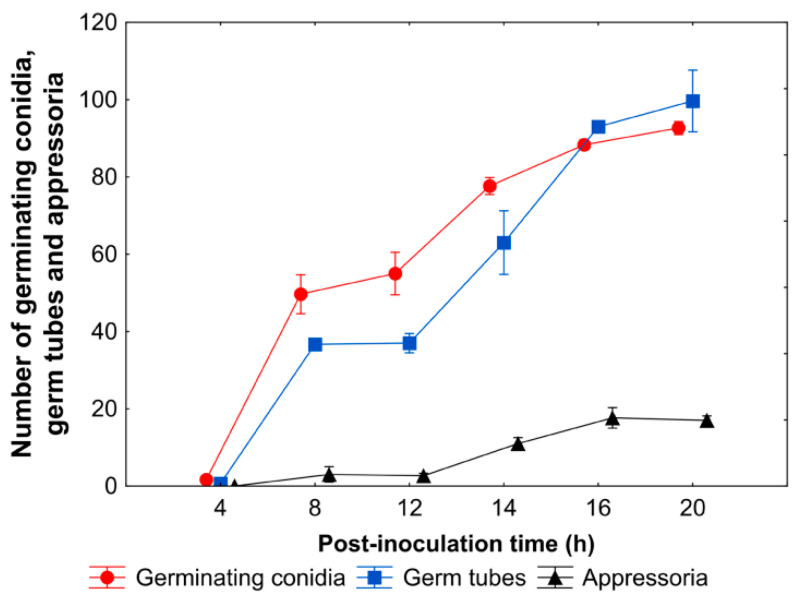
Quantitative data of *A. brassicicola* development on *B. oleracea* leaves. The values are means ± SE of 3 replicates.

**Figure 3:**
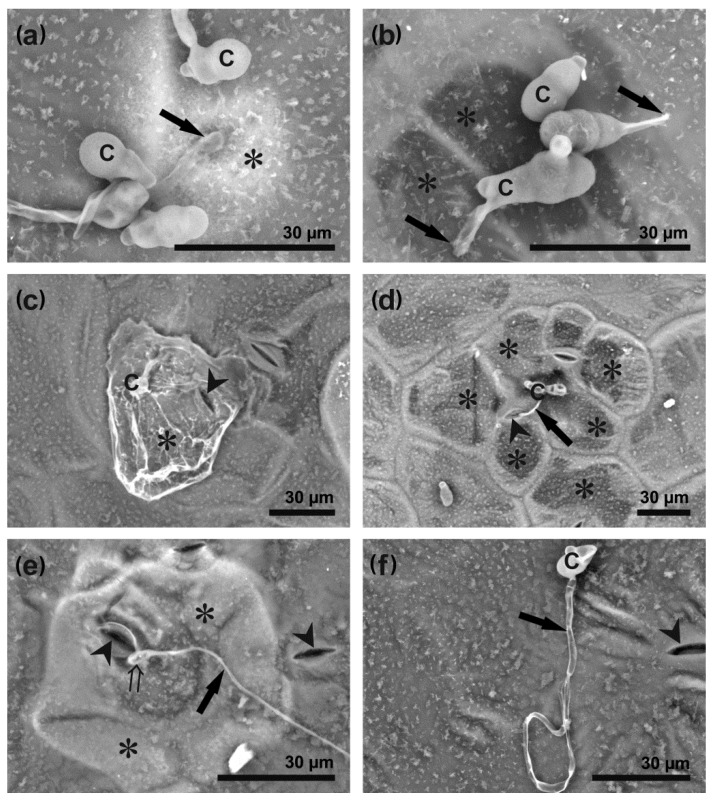
*Alternaria brassicicola* penetration modes and different host cell reactions. The images were capture using SEM at 24 hpi. (**a**) a bright ‘halo’ (asterisk) around the appressorium (arrow) penetrating the host cell; (**b**) direct penetration (arrows) caused a collapse of epidermal cells (asterisks) underneath germinating conidia; (**c**) dissolved wax layer (asterisk) around germinating conidium (arrowhead points to stoma); (**d**) germination tube (arrow) entering the leaf via stoma (arrowhead) surrounded by collapsed epidermal cells (asterisks); (**e**) germination tube (arrow) forming an appressorium (double tail arrow) over stoma (arrowhead). Neighboring epidermal cells (asterisks) have fortified cell walls; (**f**) symptomless growth of hypha (arrow) on leaf surface (arrowhead points to stoma). Abbreviation: C—conidium.

Following inoculation of *B. oleracea* leaves, conidia began to germinate at 6–8 hpi, developing germ tubes at subsequent time points, which branched and elongated into hyphae at a later stage of infection (Figure S2). Although the number of both germinating conidia and germ tubes increased gradually with the post-inoculation time flow (*p* < 0.001, r = 0.94), the number of germ tubes did not significantly increase over the number of germinating conidia at any investigated time points (Figure 2). First dome-shaped appressoria were formed at 8 hpi and their number increased in a time-dependent manner (*p* < 0.001, r = 0.86), remaining at a similar level (not exceeding 20%) from 14 hpi (Figure 2). The fungus penetrated host epidermal cells through appressoria, stomata or directly triggering various host cell reactions at the same time (Figure 3). At the beginning of penetration, a bright ‘halo’ could be observed under a scanning electron microscope (SEM) around the penetration sites, regardless of the mode of penetration (Figure 3a and Figure S3a,b). Direct penetration caused rapid disturbance and collapse of the host epidermal cells, visible as dark areas divided by light cell walls (Figure 3b), but also dissolution of the leaf wax layer (Figure 3c and Figure S3c). Although penetration through stomata caused epidermal cell disturbance and death spreading to adjacent cells (Figure 3d), host epidermal cells with fortified cell walls were also observed in this case (Figure 3e). Regardless of the mode of fungal penetration, the first brownish host epidermal cells, indicating successful penetration sites, appeared as early as 14–16 hpi (Figure S2). However, germinating conidia showing symptomless growth could also be observed on the leaf surface even at 24 hpi (Figure 3f).

**Figure 4:**
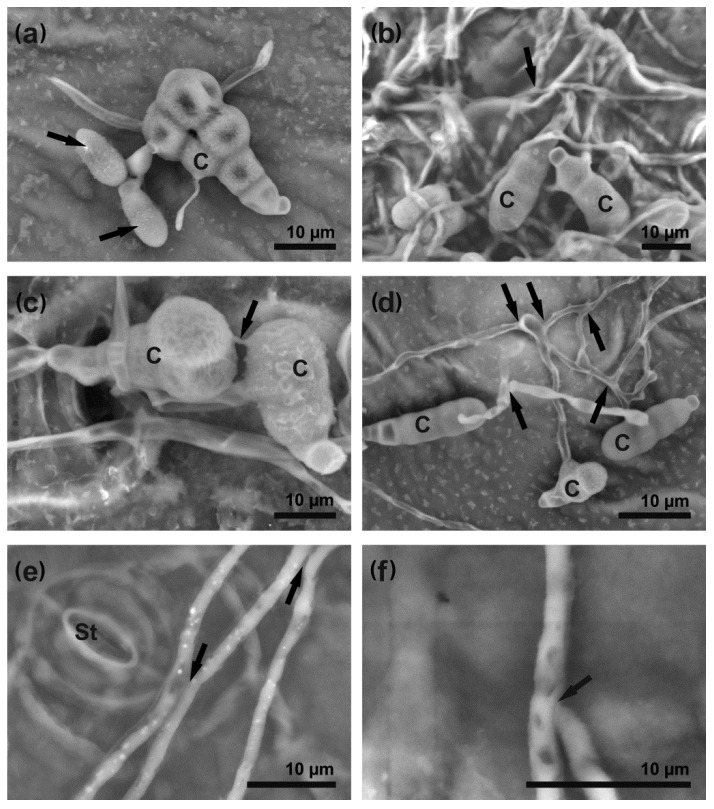
*Alternaria brassicicola* mycelium formation on *B. oleracea* leaves. The images were captured using SEM. (**a**) direct conidiation—new conidia (arrows) bud off from a mature conidium (24 hpi); (**b**) massive elongation and branching of hyphae (arrows) on the leaf surface (24 hpi); (**c**,**d**) conidial anastomosis tubes (CAT, arrows) form connections between conidia (24 hpi); (**e**) anastomoses (arrows) between hyphae (48 hpi); (**f**) fusion of hyphae (arrow) (48 hpi). Abbreviations: C—conidium, St—stoma.

New young conidia were produced by mature parental conidia (used as an inoculum) during direct conidiation, taking place between 14 and 24 hpi (Figure 4a). The formation of the conidial chain developing on short conidiophores was observed later at 48 hpi. 

The advanced stage of *A. brassicicola* development—colony formation—began as a tangle of elongating germ tubes developing into hyphae at 16–24 hpi (Figure 4b, Figures S2 and S3d). Connections between different parts of a developing colony were provided by bridges formed directly between two mature conidia or germ tubes, in the form of conidial anastomosis tubes (CATs) (Figure 4c,d) and fusions between hyphae—anastomoses (Figure 4e,f). The expanded colony caused damage and overgrew host tissues from the surface to the bottom side of the infected leaf at 48 hpi (Figure S3e–g) and sometimes chlamydospores could be observed on leaf surface at 72 hpi (Figure S3h).

### 3.3. Ultrastructure of Infected Host Cells

The ultrastructure of infected B. oleracea leaves revealed that *A. brassicicola* hyphae grew both inter-and intracellularly within the host leaf tissues (Figures S4 and S5a–d). Most of the hyphae had a regular ultrastructure, but a small number of hyphal cells with some level of degradation and osmiophilic granules were also observed (Figure S5e). In the infected mesophyll cells, the changes such as damage to the cell wall, plasmolysis and various levels of organelle disintegration, were dependent on the distance from the necrotic area and presence of hyphae (Figure S4). In the chlorotic area without hyphae, the host cell integrity was retained, although minor ultrastructural changes became evident. 

**Figure 5:**
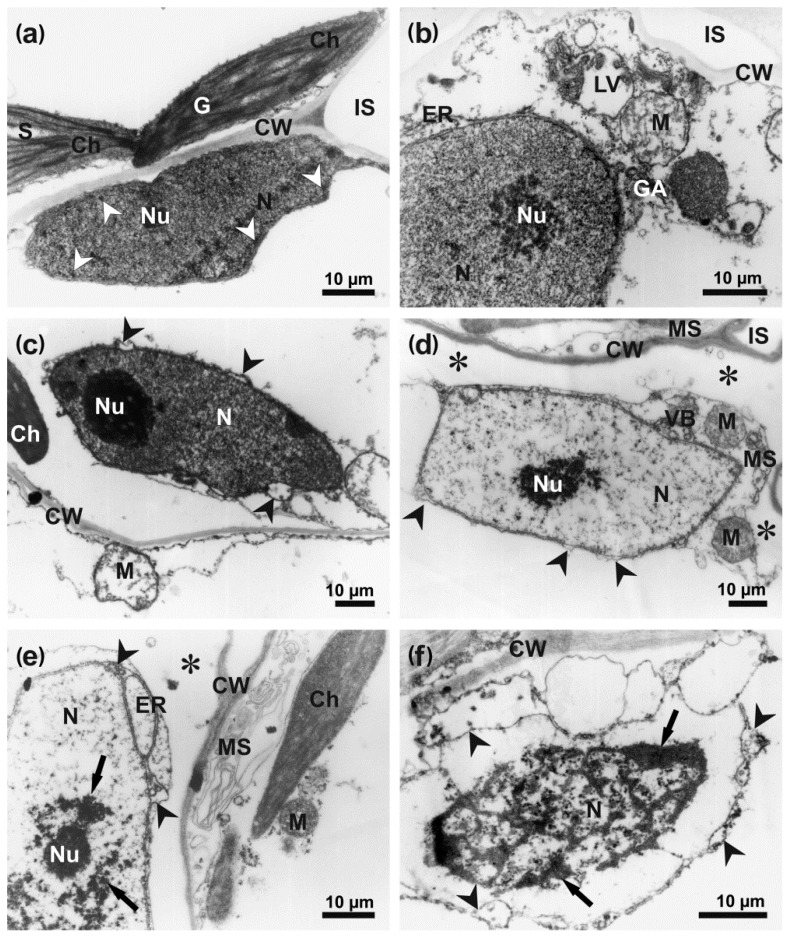
Degradation of mesophyll cells in *B. oleracea* leaf infected with *A. brassicicola* at 48 hpi. The images were captured using TEM. (**a**) a typical nucleus with a nucleolus and small heterochromatin grains (arrowheads), and chloroplasts in control mesophyll cell; (**b**) fragment of cell with degraded protoplast and nucleus with well-preserved nucleolus and fine granular chromatin; (**c**) nucleus with a nucleolus, condensed clumped chromatin and locally dilating nuclear envelope (arrowheads); (**d**) fragment of cell with degraded protoplast detached from the cell walls (asterisks), nucleus with granular chromatin, locally dilated envelope (arrowheads) and electron-dense deteriorating nucleolus; (**e**) fragment of strongly degraded cell with protoplast detached from cell walls (asterisk), pycnotic stage nucleus with osmiophilic remnants of chromatin (arrows) and dilated nuclear envelope (arrowheads); (**f**) terminal stage of nucleus degradation with clumps of chromatin (arrows), broken and dilated envelope (arrowheads). Abbreviations: Ch—chloroplast, CW—cell wall, ER—endoplasmic reticulum, G—granum, GA—Golgi apparatus, IS—intercellular space, LV—lytic vacuole, M—mitochondrion, MS—multilamellar structure, N—nucleus, Nu—nucleolus, VB—vesicular body.

**Figure 6:**
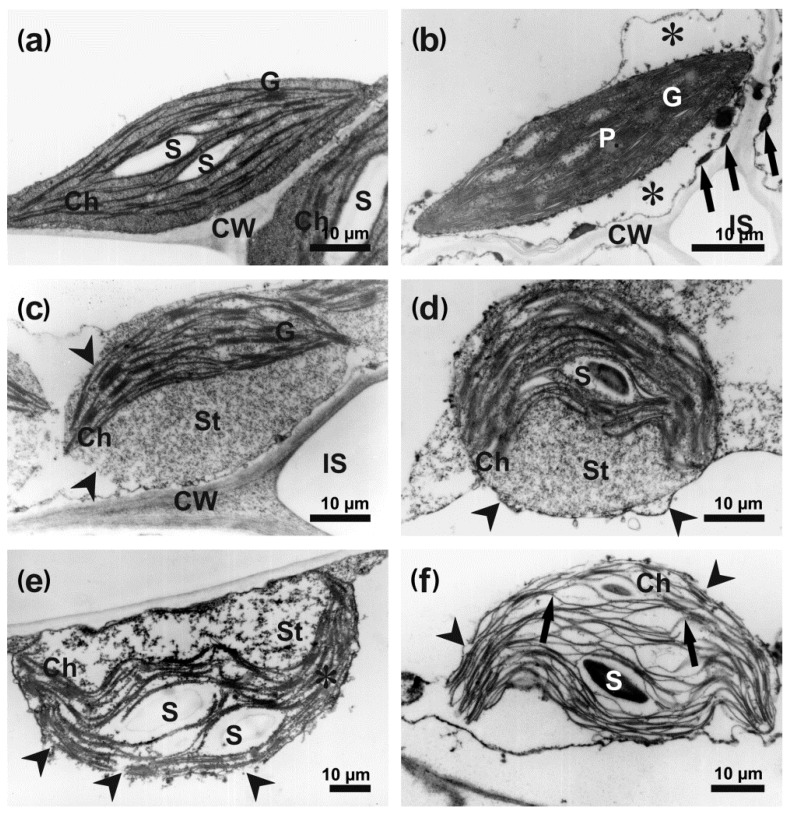
Disintegration of chloroplasts in *B. oleracea* leaf mesophyll cells infected with *A. brassicicola* at 48 hpi. The images were captured using TEM. (**a**) lenticular-shaped chloroplast with typical thylakoid and grana structure, and small starch grains in uninfected leaf; (**b**) fragment of mesophyll cell with degraded cytoplasm (asterisks), osmiophilic granules in plasmalemma (arrows) and a typical well-preserved chloroplast with plastoglobuli; (**c**) round-shaped chloroplast with a still preserved thylakoid and grana system, disintegrated chloroplast envelope (arrowheads) and swollen electron-translucent stroma; (**d**) round-shaped chloroplast with cup-shaped thylakoid system, reduced number of grana, locally swollen envelope (arrowheads) and electron-translucent stroma; (**e**) strongly disintegrated chloroplast with flocculent remnants of stroma, locally disintegrated envelope (arrowheads) and collapsed thylakoid system (asterisks) devoid of grana; (**f**) remnants of chloroplast with destroyed envelope (arrowheads) and thylakoids with widened lumens (arrows). Grana and stroma are missing. Abbreviations: Ch—chloroplast, CW—cell wall, G—granum, P—plastoglobule, S—starch grain, St—stroma.

In the control leaves, the mesophyll cells had a classical ultrastructure, with a large central electron-translucent vacuole and a narrow layer of homogeneously electron-dense cytoplasm, containing organelles, located parallel to the cell wall. Cell nuclei were surrounded by a continuous envelope perforated with nuclear pores only. Only few and small electron-dense heterochromatic grains were located next to the nuclear envelope. A well-distinguishable single nucleolus was present inside uniformly electron-dense euchromatin (Figure 5a). Lenticular chloroplasts with electron-dense stroma and a regularly arranged thylakoid and grana system contained up to three small starch grains (Figure 6a).

The mesophyll cells at the border of the chlorotic and necrotic areas, especially in the close proximity of hyphae, showed various degrees of plasmolysis and organelle disintegration. They contained numerous lytic vacuoles, membranous multilamellar structures and vesicular bodies as well as osmiophilic granules close to the cell wall and organelles (Figure 5 and Figure 6). Despite the appearance of symptoms of cell lysis, the ultrastructure of the nuclei was still well-preserved in some cells (Figure 5b), showing only local nuclear envelope swelling. However, the nucleoplasm slowly lost its electron-density and acquired a fine granular and fibrillar appearance (Figure 5c,d). In the more advanced stages of degradation, the nucleoplasm region turned strongly electron-translucent, and remnants of chromatin coalesced, forming electron-dense grains (Figure 5e,f). With the exception of the terminal stages of nucleus degradation, the remnants of nucleoli remained recognizable (Figure 5c,e) and the nuclear envelope was absent only in completely degraded cells (Figure 5f).

The degradation of chloroplasts was more spectacular and distinct, and clear stages of their disintegration could be discriminated. First of all, numerous plastoglobuli appeared in still well-preserved chloroplasts (Figure 6b). Subsequently, chloroplasts started to change their shape from lenticular to round (Figure 6c,d). This phenomenon was accompanied by the swelling of the stroma that lost its electron-density, gradual disintegration of the chloroplast envelope, and formation of the cup-shaped or bowed arrangement of the thylakoid systems (Figure 6c–e). Further degradation of the chloroplasts encompassed the final degeneration of the chloroplast envelope, disappearance of grana, distention, and dilution of the thylakoid lumens and the disintegration of the stroma (Figure 6f). Despite the level of degeneration of the lamellar system, the chloroplasts still contained starch grains or their hydrolyzed remains (Figure 6).

### 3.4. Microarray Analysis

The transcriptional profiling of *B. oleracea* leaves infected with *A. brassicicola* using 29 k Arabidopsis Oligonucleotide Microarrays was performed. Out of 29,100 microarray oligonucleotide probes, 8654 could be reliably mapped to *B. oleracea* nucleotide sequences, which represented 8014 unique genes. The expression of these genes in infected plants at 12, 24, and 48 hpi was compared to control plants (0 hpi). Significant changes in gene expression (FDR < 0.1) were observed as early as 12 hpi (27 genes) and they intensified while the infection progressed, with 189 genes regulated at 24 hpi and 410 genes at 48 hpi. Overall, 487 distinct genes were significantly affected in infected *B. oleracea* leaves (Table S1). 

**Figure 7:**
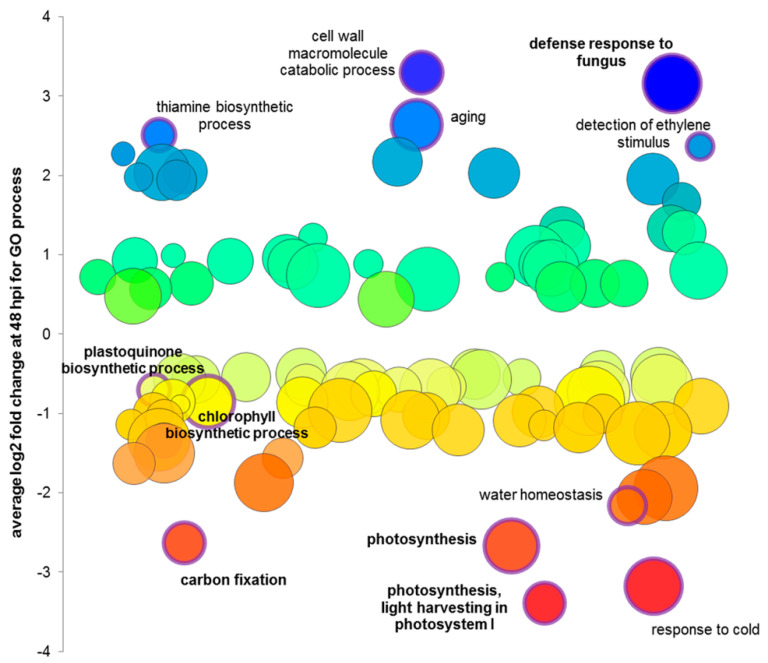
Gene Ontology of Biological Processes in *B. oleracea* leaves during *A. brassicicola* infection.

Based on the results of the statistical analysis, the Gene Ontology (GO) and MapMan analyses were performed to annotate the significantly up- and downregulated genes, and to determine the most affected biological processes and activation of individual genes during *B. oleracea* infection with *A. brassicicola*. Biotic stress response-related processes, such as the cell wall macromolecule catabolic process and defense response to fungus, were among the most extensively upregulated GO terms (Figure 7, Table S2). They were correlated to the microscopic results obtained in this study to uncover the differential responses of host cells to the infection within the inoculation site (Figure 3 and Figure 7, Tables S2 and S3). The upregulation of host genes involved in aging, detection of ethylene stimulus and thiamine biosynthesis indicated general reprogramming of the host transcriptome in response to the infection. Analysis of stress-related genes at 48 hpi revealed differential expression of 167 genes, with 87 of them, mainly involved in cell response signaling, cell wall degradation, protein degradation, auxin-induced metabolism, being downregulated. However, transcriptional activation of 80 stress-related genes, among others *LRR-RLK7* (AT1G09970) encoding leucine-rich repeat transmembrane protein receptor-like kinase 7, *WRKY33* (AT2G38470) encoding transcription factor, *PDF1.2* (AT5G44420) encoding jasmonate-responsive plant defensin or *FAH1* (AT4G36220) encoding ferulate-5-hydroxylase 1 and *CCoAMT* (AT1G67980) encoding caffeoyl-CoA 3-O-methyltransferase involved in lignin biosynthesis, even at 48 hpi, indicated that, despite the successful colonization of the susceptible host by *A. brassicicola*, some of the host cells battled with the fungus and basal defense signaling was still active (Table S4). 

**Figure 8:**
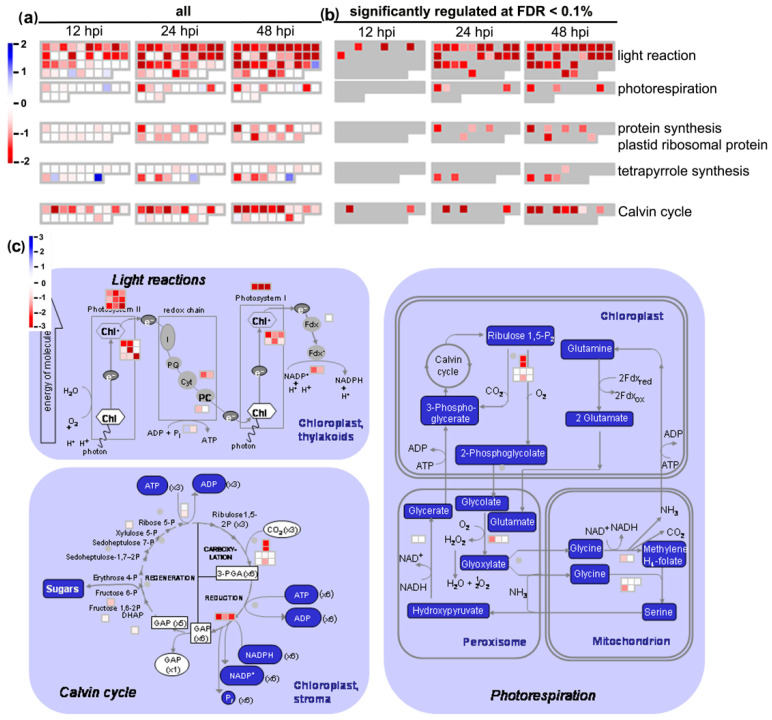
Microarray-based analysis of expression changes of photosynthesis-related *B. oleracea* genes during the course of *A. brassicicola* infection. (**a**) MapMan analysis of all mapped photosynthesis-related genes (104 genes); (**b**) MapMan analysis of photosynthesis-related genes significantly regulated at FDR < 0.1; (**c**) MapMan diagram of significant expression changes of photosynthesis-related genes at 48 hpi.

In turn, photosynthesis-related processes were strongly downregulated (Figure 7, Tables S2 and S3) in agreement with the observed severe changes in the ultrastructure of chloroplasts during infection (Figure 6). Interestingly, gradual downregulation of photosynthesis-related gene expression was observed during the entire course of *B. oleracea*–*A. brassicicola* interaction investigated in this study (Table S5). The majority of the 104 genes that were mapped to photosynthesis-related processes by the MapMan analysis, were negatively regulated at one or more time points (Figure 8a) and the number of significantly downregulated genes (at FDR < 0.1) increased from 6 at 12 hpi, through 32 at 24 hpi, up to 44 genes at 48 hpi (Figure 8b, Table S5). All crucial photosynthesis-related processes, such as light reactions, Calvin cycle, photorespiration, plastid ribosomal protein synthesis and tetrapyrrole synthesis were affected (Figure 8c). The distinctive genes in the photosynthesis light reaction category were LHCb and LHCa, encoding chlorophyll *a*/*b*-binding proteins, which are the part of photosystem II and photosystem I light harvesting complexes, respectively, and thus are important components of the chloroplast membrane system. These genes were downregulated as early as 12 hpi, with an increasing effect at subsequent time points. In the Calvin cycle, constituting the dark reaction of photosynthesis, the genes encoding the RuBisCO small subunit and glyceraldehyde 3-phosphate dehydrogenase were also downregulated at all investigated time points in infected *B. oleracea* leaves. However, changes in the expression of genes involved in photorespiration, plastid ribosomal protein synthesis and tetrapyrrole synthesis started later, at 24 and 48 hpi (Table S5). Additionally, consistent with the negative regulation of photorespiration-related genes, the downregulation of genes involved in water homeostasis was also observed (Figure 7). Remarkably, *AtCLH2* (AT5g43860) encoding chlorophyll-chlorophyllido hydrolase 2 annotated by MapMan as a tetrapyrrole synthesis-related gene was the only photosynthesis-related gene consistently upregulated at all three examined time points of infection (however, it did not pass the criteria of statistical significance) (Table S5).

### 3.5. Photosynthetic Parameters Analysis

**Figure 9:**
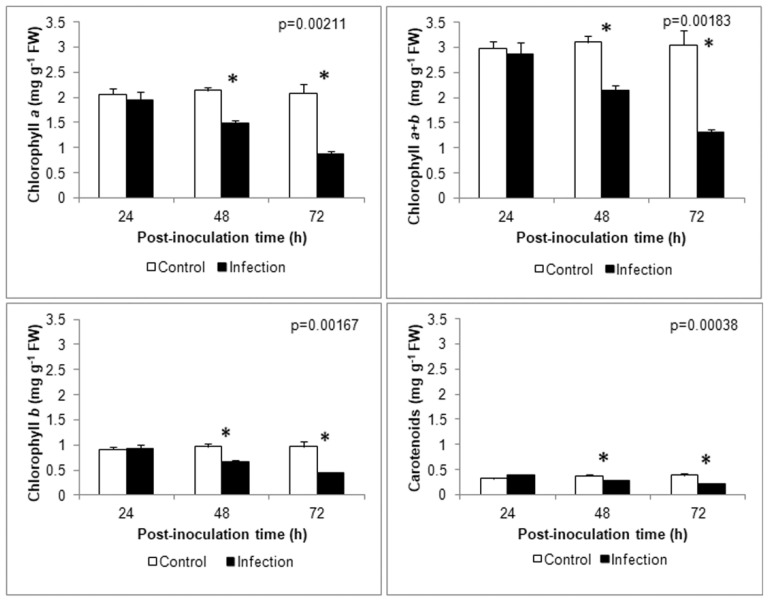
Time-course analysis of photosynthetic pigment content. The means ± SE were obtained in 3 independent experiments (*n =* 3). Asterisks indicate significant differences between control and infection at each time point according to the Duncan test (* *p* < 0.05).

Based on the results of the host cell ultrastructure and microarray data, potential changes in photosynthetic pigment content and chlorophyll a fluorescence were investigated. As suspected, the content of chlorophyll *a*, *b*, *a* + *b* and carotenoids decreased in infected leaves compared to control at 48 and 72 hpi, but no significant differences between the content of any investigated photosynthetic pigments in the control and infected leaves were observed at 24 hpi (Figure 9). 

**Figure 10:**
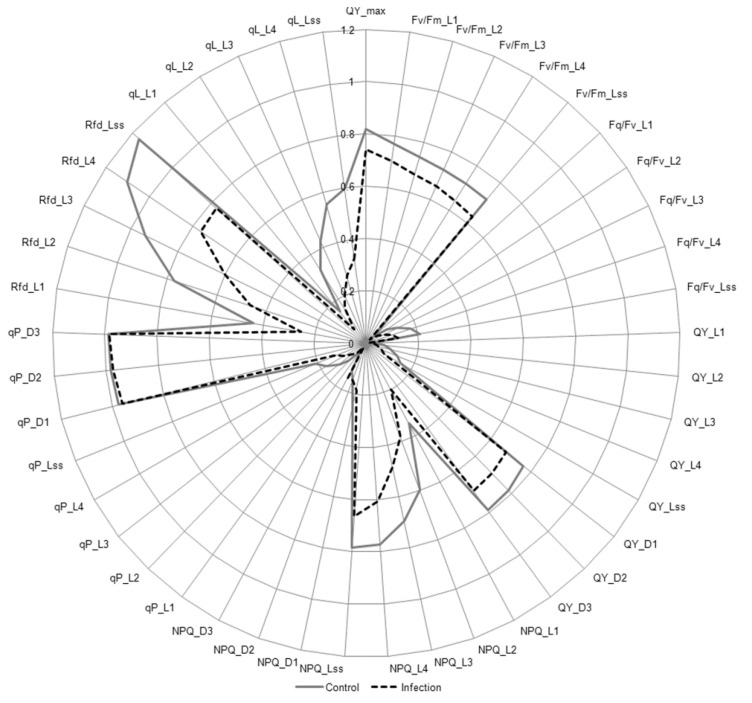
Analysis of chlorophyll *a* fluorescence parameters in *B. oleracea* leaves infected with *A. brassicicola* at 48 hpi. Values are the means obtained in 3 independent experiments (*n =* 3; detailed analysis is available in Table S6).

The analysis of chlorophyll *a* fluorescence quenching revealed that the steady-state photosystem II (PSII) quantum yield (F_v_/F_m__Lss) and the empiric parameter used to assess plant vitality (Rdf) significantly decreased in infected *B. oleracea* leaves in a time-dependent manner (Figure 10, Table S6). The significant decrease of variable fluorescence photochemical quenching (qP), PSII open centers fraction (qL), and PSII efficiency factor (F_q_/F_v_) in light adaptation periods and steady-state light during infection indicated partial blocking of electron transport to PSII reaction centers (Figure 10). The temporary increase in the value of non-photochemical quenching (NPQ) in infected leaves at 48 hpi was only visible during one of the dark relaxation periods (NPQ_D3). In turn, NPQ in light adaptation periods (NPQ_L1-L3) showed a significant decrease in infected leaves compared to the control at 24 hpi and 48 hpi (Figure 10, Table S6). Despite this, the maximum quantum yield of PSII significantly decreased in infected *B. oleracea* leaves compared to the control at 48 hpi, as evidenced by the values of QY_max and QY_Lss (Figure 10).

## 4. Discussion

Infections caused by necrotrophic fungi in host plants during susceptible interactions are related to unrestricted cell death visible as spreading lesions, the appearance of which is strictly correlated to a fungal infection cycle and the production of a wide set of virulence factors [37,38]. The cabbage cultivar ‘Glory of Enkhuizen’ used in the presented study has been considered to be susceptible to *A. brassicicola* infection under laboratory conditions and moderately resistant in the field [3]. Macroscopically visible necrotic lesions on leaves during *A. brassicicola* infection spread gradually from small brown spots at 24 hpi to larger necroses surrounded by a chlorotic area in a leaf position-dependent manner similarly as described for other Brassicas [3,9,39].

### 4.1. Timing of A. brassicicola Infection Cycle Depends on the Host Leaf Surface

In our study, the *A. brassicicola* infection cycle was delayed on the second leaf of ‘Glory of Enkhuizen’ compared to the susceptible white, red and Savoy cabbage cultivars infected with the same strain. The fungal germination began about 2 h later than on the white cabbage cultivar ‘Stone Head’; however, the appearance of the first appressoria at 8 hpi was in accordance with the previous study [11]. The delayed germination of *A. brassicicola* conidia may be related to the thicker wax layer on the ‘Glory of Enkhuizen’ leaf surface that might have caused difficulties in conidial adherence to the leaf surface and/or a delayed recognition of the host surface signals by the conidia [40,41,42]. Following germination, the fungus penetrated host epidermal cells through appressoria, stomata or directly without any preferential mode of penetration, but, on other susceptible cabbage cultivars, *A. brassicicola* penetrated the leaf surface mainly through appressoria and rarely through stomata [11]. Nowakowska et al. [3] have claimed that the cabbage cultivars were penetrated mainly directly and through appressoria, and only rarely through stomata or without any preferential mode of penetration independently of a cultivar.

### 4.2. Host Cells Respond Differentially to Penetration

Using a SEM, we found that the fungus attempts to penetrate leaf surface, regardless of the mode of penetration, were accompanied by a bright ‘halo’ formation as early as 12 hpi (Figure 3a and Figure S3), indicating that, at first, the host cell reaction to penetration was defensive. Such clear ‘halos’ around penetration sites have been observed during a resistant interaction of a biotrophic fungus *Blumeria graminis* f. sp. *tritici* on a wheat cultivar carrying effective resistance genes and also on a susceptible one, using cryoscanning electron microscopy [43]. Possibly, these ‘halos’ were described as papillae formed around penetration sites of *A. brassicicola* on the leaves of both susceptible and moderately resistant cabbage cultivars after double staining with trypan blue and aniline blue using confocal laser scanning microscopy [3]. The biochemical analysis revealed that the ‘halo’ contains callose, phenolic compounds and an elevated level of calcium ions [43]. The ‘halo’ phenomenon indicates that the host cells are trying to combat fungal invasion at this point of the susceptible interaction and stay alive. It also shows that the *A. brassicicola* infection cycle probably contains a very short biotrophic phase, although there is a general agreement that a necrotrophic fungus first kills a host cell by secreting toxins, and then invades it. Interestingly, it has been postulated that another necrotrophic fungus, *B. cinerea*, should be considered as a hemibiotroph having a short biotrophic phase. *Botrytis cinerea* suppresses early host defense reactions by the secretion of small RNAs (sRNAs), thus leading to the silencing of host genes, but, to achieve this, the host cell must be alive [44]. It has been also shown that during invasion and the establishment of a necrotrophic interaction, *B. cinerea* ‘sacrifices’ many of its invading hyphae by subjecting them to cell death induced by plant-secreted cell death inducing factors, to get a chance to release fungal cell death inducing factors by the surviving hyphae into plant cells [45]. It could also be a mechanism used by *A. brassicicola* and an explanation for the presence of hyphae with deteriorating protoplasts (Figure S5e). 

At later stages of an *A. brassicicola* infection (from 12–16 hpi), the successful penetration sites appeared on SEM images as electron-dense collapsed epidermal cells (Figure 3b) or brownish possibly dead cells under bright field light microscopy (Figure S2), which have been previously described during *A. brassicicola* infection [11]. Brownish cells are also frequently found at penetration sites of other necrotrophic as well as biotrophic fungi [46]. Browning of successfully infected epidermal cells may have been caused by the activation of peroxidases and phenolics within the cell wall and/or a production of ROS by dying host cells, indicating the suppression of host cell defense reactions and a highly susceptible response at this stage of infection [11,22,47,48].

### 4.3. A. brassicicola Colony Formation Is Triggered by Successful Penetration

Successful penetration of the susceptible host epidermal cells by *A. brassicicola* was a signal triggering colony formation at inoculation sites. Connections between different regions of an expending mycelial network were established via CATs and anastomoses (Figure 4). Their formation is a typical feature of many filamentous fungi [49,50], but these phenomena are mostly described when fungi grow in media rather than *in planta* during infection. CATs are usually formed during the initiation of a colony, and facilitate the transport of water and nutrients as well as horizontal gene transfer [51,52]. Similarly to CATs, the formation of fusions between hyphae (anastomoses), originating from the same or different conidia allows them a proper distribution of nutrients, transduction of chemical signals, and even exchange of genetic material [53]. Moreover, anastomosis formation is a prerequisite for *A. brassicicola* virulence, as indicated by the development of an *A. brassicicola aso1* mutant that is unable to form anastomoses, and, when tested in planta, appears to be unable to spread beyond the inoculation site [54].

### 4.4. Changes in Host Cell Ultrastructure and Transcriptome Reprogramming

The appearance of necrotic spots indicated successful invasion and colonization of the host tissues by *A. brassicicola*, although host cells were differentially affected within the inoculation site depending on the distance from the invading hyphae, as evidenced by the gradual degradation of organelles. Similar individual changes in the ultrastructure of infected plant host cells, such as cell lysis, disintegration of the nuclei and chloroplasts, or the presence of osmiophilic granules have been observed in other pathosystems during infection with viruses, bacteria, and fungi [55,56,57,58]. It has to be mentioned that rounded chloroplasts were also observed in *B. juncea* cells in response to *A. brassicicola* infection, although the cup-shaped thylakoid system was not found [9]. Changes of chloroplast shape and location of thylakoids could be related to cell lysis and, as a consequence, changes of osmotic pressure of stroma could have occurred. However, the underlying processes behind chloroplast degradation during infection are complex and require further extensive investigation. In the case of infected *B. oleracea* mesophyll cells, changes of organelle ultrastructure could be a result of the action of toxins and secondary metabolites secreted by *A. brassicicola* [16] and general reprogramming of the host transcriptome and metabolome in response to the infection [59,60]. *Alternaria* species secrete various non-host-(nHSTs) and host-selective toxins (HSTs) which are responsible for the degradation of different organelles within an infected cell in over 200 plant species. For example, *Alternaria alternata* pathotypes produce different HSTs depending on the plant species, such as the AM-toxin that degrades the plasma membrane and chloroplasts in apple; the ACR-toxin that causes damage of the mitochondria in lemon; the AK-toxin, AF-toxin and ACT-toxin that target the plasma membrane in susceptible cultivars of Japanese pear, strawberry and tangerine, respectively [61,62,63]. However, a possible effect of identified *A. brassicicola* secreted compounds during infection on plant cell structures or their influence on the host transcriptome has not been described yet. It has to be emphasized that visualized differential *B. oleracea* cell responses to *A. brassicicola* infection, from defensive to highly susceptible, were confirmed by our microarray results (Table S4), and were also concordant with the previous studies on plant gene expression during infection of susceptible *B. oleracea* and *B. napus* with *A. brassicicola* [64,65]. The most upregulated categories revealed in the analysis of Gene Ontology of Biological Processes were aging, the detection of ethylene stimulus, thiamine biosynthetic processes, cell wall macromolecule catabolic processes and defense response to fungus. Additionally, analysis of stress-related genes revealed upregulation of the individual genes associated with hormone metabolism (brassinosteroids, ethylene and salicylic acid biosynthesis), peroxidases, glutathione S-transferases and secondary metabolism connected to flavonoid biosynthesis (Table S4). Many of these genes have been previously described as important factors in general plant cell response to biotic stress and identified in transcriptome profile analyses of other susceptible hosts infected with *Alternaria* species [66,67]. Some of these genes undoubtedly are characteristic for the *B. oleracea* response to infection by *A. brassicicola* [59,64], albeit confirmation of their roles requires further investigations based on comparative transcriptomics and proteomic and metabolomic approaches. It has to be emphasized that the up- and downregulation of the host genes in response to *A. brassicicola* revealed in our microarray analysis is concordant with a general scheme of transcriptomics response of a host to necrotrophic pathogens. Perception of necrotrophic fungi activates hormone-dependent and hormone-independent signaling pathways [68]. In resistant interactions, a hormone-dependent response to necrotrophs is mainly based on induction of genes associated with JA and ET-dependent signaling pathways to restrict pathogen spread. In susceptible interactions, genes involved in abscisic acid (ABA), brassinosteroid (BR) and auxin-dependent signaling are also often induced [67,69,70]. Regardless of the mode of interaction, hormone-independent signaling is mediated by induction of genes involved in biosynthesis of phytoalexins and pathogenesis-related proteins (PR) [68]. If a pathogen is unable to detoxify host-specific phytoalexins, they often restrict its growth as in the case of Arabidopsis and *A. brassicicola* interaction [23]. In addition, PR proteins are plant species-specific and play important roles in a pathogen recognition processes by a host as well as a host defense response. Transcriptomics analysis of tomato genotypes contrasting in response to *A. solani* infection revealed differential expression of PR protein-encoding genes. In resistant tomato genotype, the most of identified PR genes was extremely upregulated, whereas, in a susceptible genotype, the majority of these genes was downregulated [66].

### 4.5. Defense-Related Genes Are Activated Also in Susceptible Interaction

The upregulation of several stress-related genes involved in the defense response to fungus in the examined susceptible cultivar of *B. oleracea* even at 48 hpi, when necrotic lesions were fully developed, indicates that some of the host cells still attempted to combat the invasion of the fungus. The fungal hyphae spreading beyond inoculation site, even at a later stage of infection, apparently induced host defense response through PTI (pattern-triggered immunity) [59]. Probably, the perception of DAMP (damage-associated molecular pattern, e.g., products of the *A. brassicicola* cell wall degrading enzymes or plant secreted peptides) or PAMP (pathogen-associated molecular pattern, e.g., chitin) through a PRR (pattern recognition receptor) triggered the PTI-associated signaling cascade (albeit transcription of MEK1 was negatively regulated) and activated WRKY33 in infected *B. oleracea* cells, similarly to the situation observed in Arabidopsis during fungal infection [71]. Interestingly, the only upregulated gene encoding PRR in our microarray analysis was *RLK7* (Table S4). RLK7 belongs to the category XI of RLKs and acts as a receptor for PIP1 secreted peptide. Elevated expression of *PIP1* has been described in Arabidopsis guard cells during *Pseudomonas*-induced PTI, and thus the peptide plays a role in stomatal immunity [72]. Moreover, RLK7 also contributes to Arabidopsis resistance to *B. cinerea* [73] and *Phytium irregulare* [74]. However, *LYK5*, the other gene encoding RLK, which is an important receptor engaged in chitin perception in Arabidopsis [75], was downregulated. The upregulated genes involved in defense signaling such as *WRKY33* [76] and *PDF1.2* [77] have been identified in *A. thaliana* signaling pathways and are required for signaling resistance to necrotrophic fungi *A. brassicicola* and *B. cinerea*. In Arabidopsis, WRKY33 transcription factor is responsible for the activation of camalexin biosynthesis [78], which efficiently inhibits development of necrotrophic fungi. Possibly, WRKY33 activates *de novo* biosynthesis of *B. oleracea* fungus-induced phytoalexins, such as brassinin, which can be metabolized by *A. brassicicola*, and thus the fungus suppresses the first line of host defense [79]. Subsequently, JA-dependent signaling, which is characteristic for host cells infected by fungal necrotrophs, activates transcription of JA-responsive defense genes encoding PR (pathogenesis-related) proteins such as PDF1.2 involved in resistance against *B. cinerea* [80] and *A. brassicicola* [23]. However, the downregulation of *LOX2* chloroplast lipooxygenase required for JA accumulation may be also responsible for the impaired defense of the host and, in turn, salicylic acid-dependent genes become activated, which promote host cell death [81]. Moreover, local fortification of the host cell walls microscopically observed within inoculation site in our study was reflected in the upregulation of several stress-responsive cell wall and secondary metabolism-related genes, such as *FAH1* involved in lignin biosynthesis and an important component of *A. thaliana* resistance mechanism to *B. cinerea* [82] and *CCoAMT*, which is involved in the biosynthesis of cell wall-bound phenolics and lignin [83]. The strengthening of host cell walls through lignin biosynthesis and its deposition at a pathogen entry site also constitutes the first line of the host defense reaction in order to slow down or restrict pathogen development, especially as lignin is not metabolized by most pathogens [84,85]. Thus, even successful invasion of host tissues within inoculation sites does not signify that every single host cell immediately surrenders to a fungal invader. However, downregulation of numerous stress-related genes found in our study indicates that *A. brassicicola* effectively overcomes the host arsenal of defenses (Table S4). Although it has been reported that *A. brassicicola* secretes phytotoxin brassicicolin A, histone deacetylase inhibitor depudicin, siderophore N,N-dimethylcoproge [15,17], a proteinaceous host-specific toxin—AB-toxin [86,87] and many low molecular weight secondary metabolites e.g., brassicenes A to F [88], there is not yet any known putative *A. brassicicola* effector(s) targeting also still unknown *Brassica* receptor(s) and triggering ETS (effector-triggered susceptibility) or ETI (effector-triggered immunity), albeit many research groups all over the world work on *A. brassicicola* pathogenicity factors [16]. 

### 4.6. Downregulation of Photosynthesis Is Probably Not Only a Part of Susceptible Interaction

Furthermore, we have focused on changes in the ultrastructure of chloroplasts in infected mesophyll cells, mostly due to the observed clear stages of their degradation (Figure 6) and the fact that analysis of our microarray data pointed out photosynthesis as the most negatively regulated process during infection of *B. oleracea* leaves with *A. brassicicola* (Table S3). Chloroplasts are energy and carbon source organelles, and play an important role in plant immunity as a compartment for ROS generation and the production of phytohormones, secondary metabolites, and their precursors [89]. As sensors of environmental changes, chloroplasts can shape nuclear gene expression and activate defense responses through redox flux [90]. Moreover, many pathogen-derived effectors target chloroplast-localized proteins, including components of the photosynthetic electron transport chain [91]. Therefore, changes in chloroplast ultrastructure are often used as good indicators of biotic/abiotic stress [92]. 

Gradual degradation of the chloroplast membrane system, such as widening of the thylakoid lumens and the disappearance of grana observed in infected *B. oleracea* leaves, indicated the suppression of photosynthesis light reactions and potential damage of light harvesting complexes and reaction centers. As a result, chlorophyll and carotenoid content and photosynthesis efficiency at a physiological level were decreased. Ultrastructural changes of the stroma, from its swelling to disintegration, suggested downregulation of the photosynthesis dark reaction and a decrease of carotenoid content. In severely degraded cells, the thylakoid system was still to some extent preserved, whereas the chloroplast envelope and stroma were totally disintegrated (Figure 6f). The observed changes in the chloroplast ultrastructure were associated with changes in the expression of photosynthesis-related genes and the physiological response of the host cells. Early downregulation of photosynthesis-related genes could be a result of the host cell defense strategy related to the shift from photosynthesis to non-assimilatory metabolism, as observed during plant infection by various pathogens, or an effect of action of an unknown *A. brassicicola*-secreted effector protein [93]. At later stages of infection (24 and 48 hpi), more genes involved in light reactions were downregulated. Fluorescence decline ratio in steady-state (Rfd) and a significant decrease in steady-state PSII quantum yield (F_v_/F_m_Lss) in infected *B. oleracea* leaves indicate inhibition of electron transport in light-dependent reactions. Confirmation of this was the decrease in the value of F_q_/F_v_, qL, as well as the qP parameter, which describes the level of energy transferred to the reaction centers and informs about the proportion of open PSII reaction centers (Figure 10) [94,95]. The reduction of photosynthesis, determined by a decrease in the effective quantum yield of PSII, has been previously observed in the interaction of *B. juncea*–*A. brassicicola* [9] and in other plants infected with necrotrophic fungi [96,97]. In addition, in wheat plants infected with *Bipolaris sorokiniana*, a decrease in F_v_/F_m_ correlating with the loss of chlorophyll has been noted [98]. 

In severe stress caused by *A. alternata* in rice, a decrease in the electron transport rate was correlated with an increase in non-photochemical quenching (NPQ) [99]. An early increase in NPQ took place following a decline in photosynthetic electron transport activity. A similar PSII response was also observed in plants infected with viruses, e.g., Pepper mild mottle virus (PMMoV) infecting pepper leaves [100]. However, such an increase can be the result of both: protective processes of PSII and damage to the photosystem [84]. In infected *B. oleracea* leaves, we generally observed a decrease in NPQ (Figure 10), which indicates a reduction in the dissipation efficiency of excess excitation energy as heat. However, it should be mentioned that not all processes associated with non-photochemical quenching lead to an increase in NPQ [101]. The decrease in NPQ in infected *B. oleracea* leaves may be due to reduced light absorption, e.g., as a result of the destruction of chloroplasts and a decrease in chlorophyll content (Figure 9), rather than a lack of thermal dissipation. In addition, in other plants (i.e., rice, tomato) exposed to necrotrophic fungi, a significant decrease in NPQ was observed both in the necrotic zone and in adjacent areas of the leaf blade, without significantly reducing the photosynthesis efficiency (measured as F_v_/F_m_) [97,102]. 

In the case of progressive degradation of chloroplasts at the inoculation site, it is difficult to expect discrete changes in the photosynthesis light reactions. Therefore, the significant decrease in the values of chlorophyll a fluorescence quenching parameters is not surprising. However, a slight decrease in QY_max and QY_Lss (Figure 10) suggests that the destruction of the photosynthetic apparatus did not completely block the electron transfer in PSII in the analyzed leaf area. It is probable that the chloroplasts of cells that have not been invaded by the fungus allow photochemical reactions to occur, although it is known that even *Alternaria* spp. metabolites alone can cause a reduction or complete inhibition of the electron transport chain from QA to QB [103,104]. This is due to competition between the toxin and QB for a binding site in the D1 protein on the thylakoid PSII membrane. In general, the photosynthetic yield of plants infected with necrotrophic fungi presents complex spatial and temporal patterns, depending on the degree of colonization the individual regions of a leaf blade by the pathogen [105].

## 5. Conclusions

Our results show that initial stages of a susceptible interaction between *B. oleracea* and *A. brassicicola* are complex, not uniform within an inoculation site, and host cell defenses are still active even at later stages of infection (48 hpi). Analysis of the microarray data suggested the possible involvement of putative genes encoding LRR receptor-like kinases in PTI in this pathosystem. Ultrastructural, molecular, and physiological analysis of infected leaves revealed photosynthesis as the most downregulated process from the onset of the infection. This finding should be taken into consideration in further research and work on a strategy for the management of black spot disease.

## Supplementary Materials

The following are available online at https://www.mdpi.com/2073-4409/9/10/2329/s1, Figure S1: Leaf position-dependent necrosis formation on *B. oleracea* leaves during *A. brassicicola* infection, Figure S2: Bright field light microscopy images depicting germination and development of *A. brassicicola* on *B. oleracea* leaf surface, Figure S3: Scanning electron microscopy (SEM) images depicting formation of *A. brassicicola* mycelium on abaxial side of *B. oleracea* leaf, Figure S4: Selected light microscopy images taken from semi-thin sections of control and infected *B. oleracea* leaves, Figure S5: Transmission electron microscopy micrographs depicting ultrastructural details of *A. brassicicola* hyphae growing inside the host leaf mesophyll, Table S1: Statistical analysis of microarray data, Table S2: Gene Ontology of Biological Processes at 48 hpi significant with FDR<0.1 expressed in *B. oleracea* during *A. brassicicola* infection, Table S3: Gene Ontology enrichment analysis, Table S4: Microarray-based analysis of stress-related genes significantly expressed during *B. oleracea* infection by *A. brassicicola* at 48 hpi (MapMan analysis), Table S5: Microarray-based analysis of photosynthesis-related *B. oleracea* genes differentially expressed during *A. brassicicola* infection (MapMan analysis), Table S6: Detailed statistical analysis of measured and calculated chlorophyll a fluorescence parameters in *B. oleracea* leaves infected with *A. brassicicola*. The microarray gene expression data generated during the current study is available in the Gene Expression Omnibus (GEO) repository under experiment number GSE155051 (https://www.ncbi.nlm.nih.gov/geo/query/acc.cgi?acc=GSE155051).
