# Supplementary material for: Complexity of Brassica oleracea–Alternaria brassicicola Susceptible Interaction Reveals Downregulation of Photosynthesis at Ultrastructural, Transcriptional, and Physiological Levels
Source: Cells. 2020 Oct 20;9(10):2329. doi: 10.3390/cells9102329 (PMC7593931; doi:10.3390/cells9102329)
Supplement: Supplementary file 1 [file cells-09-02329-s001.zip › Supplementary file Figure S1-S5.docx]

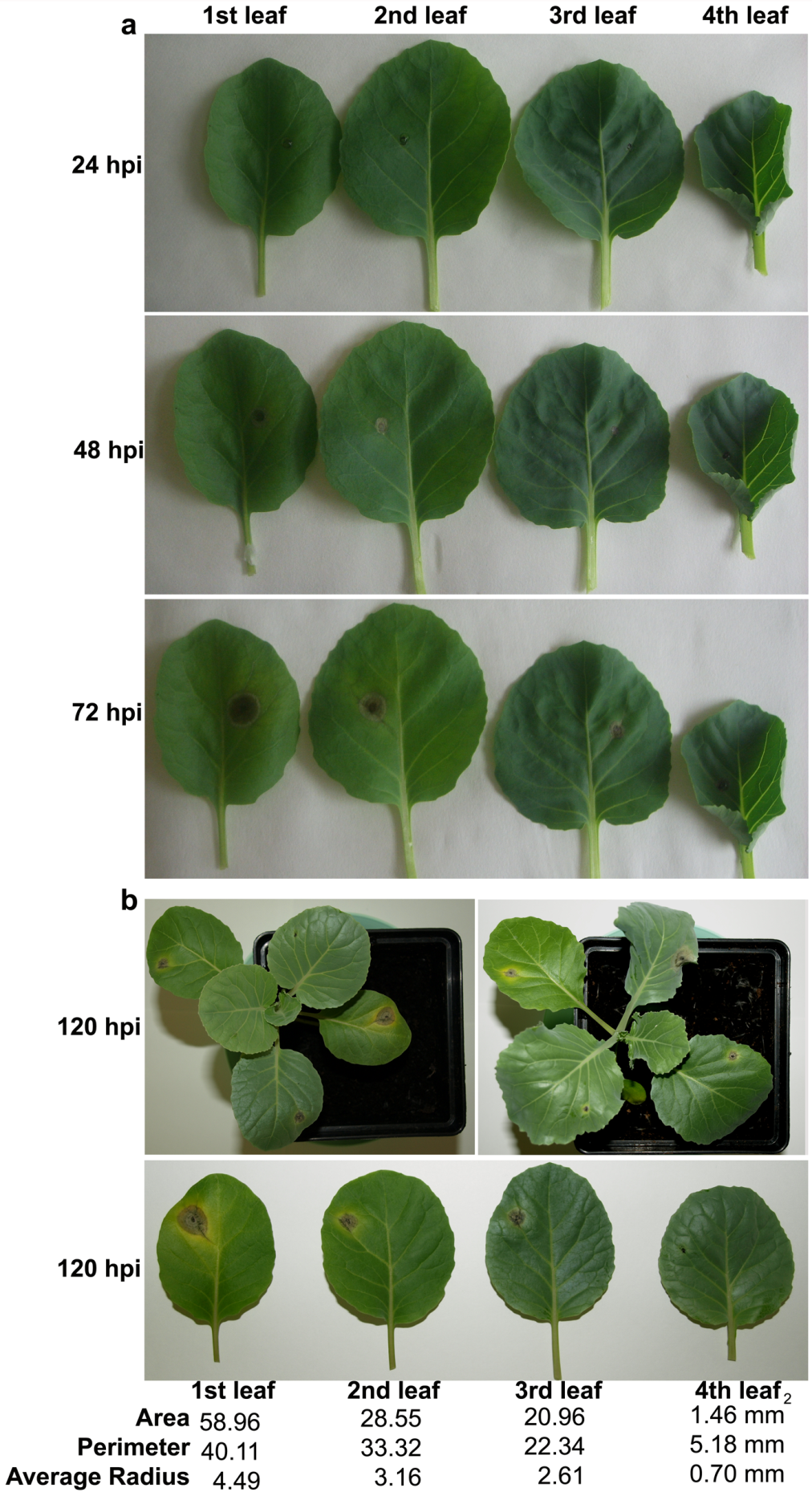


**Figure S1.** Leaf position-dependent necrosis formation on *B. oleracea* leaves during *A. brassicicola* infection.

The first leaf is the oldest one. **a** assay on drop-inoculated detached leaves from one plant with visible leaf position-dependent differences in necrosis size; **b** examples of drop-inoculated leaves in planta with leaf position-dependent necrosis parameters at a later stage of infection (120 hpi).


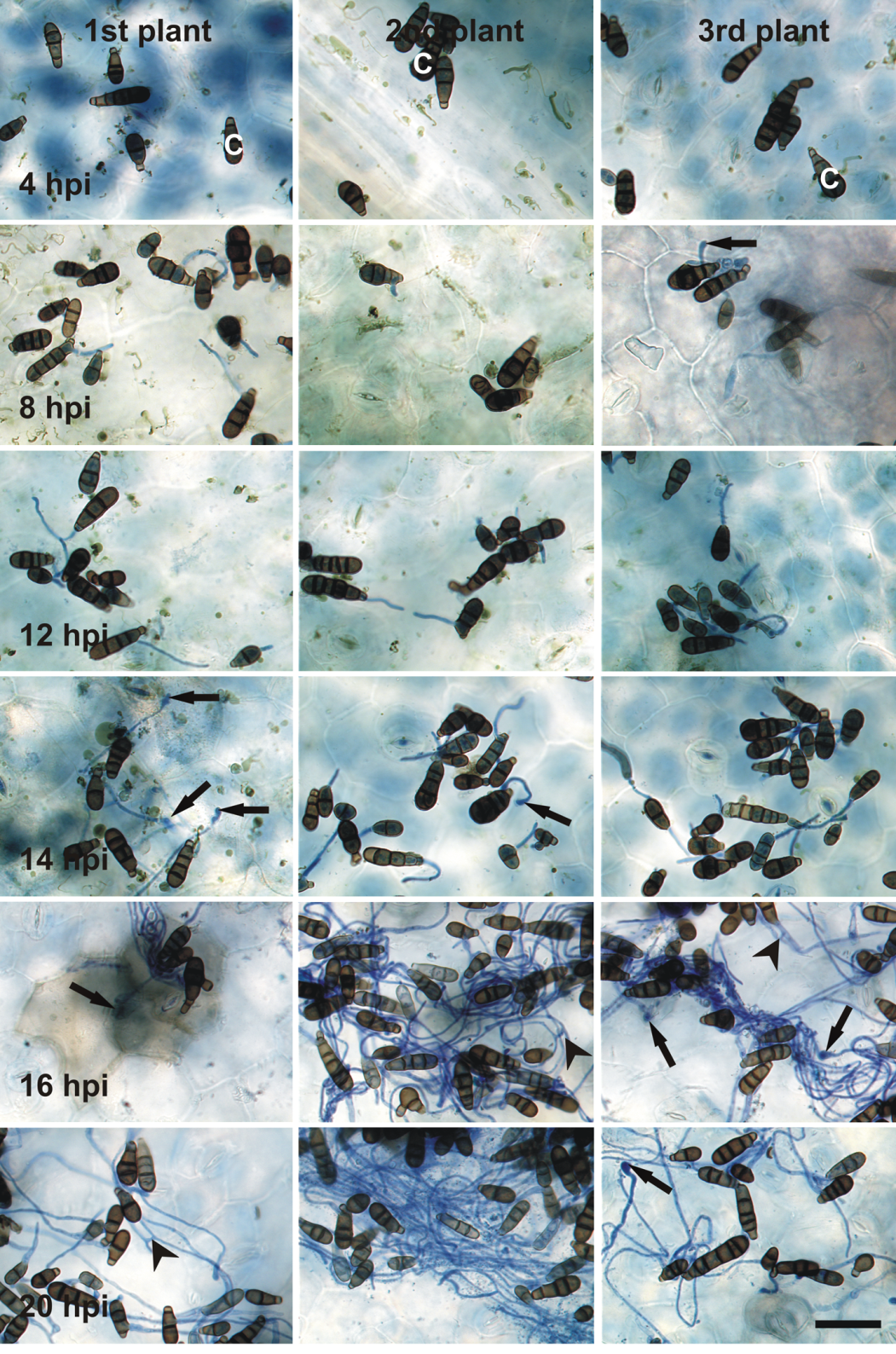


**Figure S2.** Bright field light microscopy images depicting germination and development of *A. brassicicola* on *B. oleracea* leaf surface.

Images of conidia (C) were captured from inoculation areas on the second leaf. Three plants were inoculated per time point. Arrows indicate appressoria; arrowheads indicate anastomoses. Scale bar 20 μm.





**Figure S3.** Scanning electron microscopy (SEM) images depicting formation of *A. brassicicola* mycelium on abaxial side of *B. oleracea* leaf.

**a** bright 'dots' (arrows) at the inoculation area (12 hpi); **b-f** stages of mycelium formation (arrows point to conidia, arrowheads point to hyphae) and degradation of leaf epidermal tissue (24 hpi); **g** mycelium formation (arrowhead indicates hyphae) on the adaxial side of

the leaf (48 hpi); **h** chlamydospores (arrows) on leaf surface. Scale bars 100 μm.





**Figure S4.** Selected light microscopy images taken from semi-thin sections of control and infected *B. oleracea* leaves

Samples were harvested at 48 hpi and sections (1 μm thick) were stained with toluidine blue. Images were captured under bright field light microscope and converted into greyscale mode. **a-c** sections taken from control leaf; **d-f** sections taken from chlorotic area neighboring the necrosis; **g-i** sections taken at the border of the necrosis. **a, d** and **g** cross sections of leaf lamina; **b, e** and **h** cross sections of palisade parenchyma; **c, f** and **i** cross sections of spongy parenchyma. Abbreviations: Ep - epidermis, PP - palisade parenchyma, SP - spongy parenchyma. Arrows point to selected hyphae. Scale bar 10 μm.





**Figure S5.** Transmission electron microscopy micrographs depicting ultrastructural details of *A. brassicicola* hyphae growing inside the host leaf mesophyll

**a** and **b** sections of hyphae growing in intercellular spaces; **c** and **d** sections of hyphae growing inside host cells; **e** section of degraded hypha with osmiophilic granules deposited in plasma membrane. Abbreviations: DC - degraded plant cell, fCW - fungal cell wall, IS - intercellular space, M - mitochondrion, N - nucleus, Nu - nucleolus, pCW - plant cell wall, PR - plant protoplast remnants, V - vacuole. Scale bars 10 μm.
